# The impact of precise feedback in AI vocal courses on learning outcomes: a chain mediation effect of skill mastery and self-efficacy

**DOI:** 10.3389/fpsyg.2026.1811130

**Published:** 2026-07-20

**Authors:** Ronghua Wu, Jie Wen

**Affiliations:** Fuzhou University, Fuzhou, China

**Keywords:** AI-driven precise feedback, chain mediation model, perceived skill mastery, self-efficacy, vocal learning outcomes

## Abstract

**Introduction:**

Policies such as the “New Generation Artificial Intelligence Development Plan” emphasize the application of AI across the educational process, while the multidimensional nature and psychological mechanisms of learning outcomes in vocal education remain insufficiently explored.

**Purpose:**

This study explores the impact of precise feedback in AI vocal courses on learning outcomes and investigates the chained mediating roles of skill mastery and self-efficacy to uncover the underlying psychological mechanisms.

**Methods:**

A pre-post experimental design with a control group was implemented, recruiting 114 undergraduate music education majors who were randomly assigned to either an experimental group (receiving AI-based precise feedback courses) or a control group (receiving traditional instruction). Real-time feedback was delivered through the VocalCoach Pro 3.0 system, with data collection using the Multidimensional Vocal Learning Outcomes Scale, Skill Mastery Questionnaire, and Self-Efficacy Scale. Data analysis involved *t*-tests, correlation analysis, and Bootstrap mediation effect testing.

**Results:**

AI-powered precision feedback significantly improved vocal learning effectiveness (posttest *M* = 4.271 for the experimental group vs. *M* = 3.851 for the control group; Cohen’s *d* = 1.638, *p* < 0.001). Both perceived skill mastery (mediation effect = 0.280) and self-efficacy (mediation effect = −0.145) significantly mediated the relationship, establishing the chained mediation pathway: “AI feedback → perceived skill mastery → self-efficacy → learning effectiveness” (effect size = −0.103, 95% CI [−0.310, −0.016]). The direct effects were not significant.

**Conclusion:**

AI-driven precise feedback influences learning outcomes through a chained mediating mechanism involving perceived skill mastery and self-efficacy, emphasizing the importance of the “cognitive–motivational” sequential process. This study provides theoretical foundations and practical insights for optimizing AI-based vocal music courses.

## Introduction

In recent years, artificial intelligence has gradually moved from a supporting role to a more central position within the field of education, especially in disciplines that rely heavily on skill development. Vocal training is a representative example. For a long time, it has depended largely on subjective judgment, and feedback is often delayed, which can limit learning efficiency. With the introduction of AI-based systems, this situation is beginning to change. Learners can now receive immediate and more detailed evaluations that consider multiple aspects of their performance.

This trend is also reflected in broader educational policies. Existing national development plans have highlighted the importance of smart education and its potential to support more individualized learning paths (Shi, 2022). However, simply introducing new technology does not automatically explain how learning is improved. A more fundamental issue lies in understanding how learners respond to this type of feedback from a psychological perspective.

Although it is reasonable to expect observable improvements in performance, the underlying process may be more complex. In particular, changes may occur through a sequence of internal mechanisms. Learners first interpret feedback and form judgments about their own level of skill, and these perceptions may then influence their willingness to engage with future learning tasks. In other words, the development of perceived competence and the growth of self-efficacy could play a crucial role in shaping outcomes.

Based on this consideration, the present study focuses on this relatively overlooked pathway. It examines how AI-generated precise feedback influences vocal learning results and further explores whether this influence operates through the sequential mediating effects of perceived skill mastery and self-efficacy.

In theory, the research into how AI-based accurate feedback can affect learning outcomes can be used to expand the scope of the application of the technology-enhanced learning theory to arts education, as well as deepen the theoretical approach of the feedback theory in the context of AI-facilitated learning. In practice, the present study will offer objective data on how one can design and optimize AI-based courses in the field of vocal education, thus securing the process of digitalization of vocal education. Importantly, in skill-based artistic education, locales of learning are manifested not just in behavioral manifestation of performance but also in psychology. Among the dimensions, perceived mastery of skill and self-efficacy among them are considered two important psychological constructs to evaluate the effectiveness of deep learning (An and Guo, 2024; Zhao and Liu, 2022). Thus, this research will not only consider the direct impact of generated precision feedback through AI but also attempt to clarify its indirect impact on learning activities through the mediating variables of skill mastery and self-efficacy. Explaining this intervening process is of great importance to comprehend the underlying psychological processes in the acquisition of skills in AI-assisted learning conditions.

### The impact of precise feedback in AI vocal courses on learning outcomes

Multidimensional indicators are usually used to describe the learning outcomes related to vocational education, such as the level of skill enhancement, persistence in learning, ability to transfer the acquired knowledge, and satisfaction with learning (CAO, 2024; Yin, 2024). In conventional methods of voice training, instructors usually experience some lack of objectivity because their subjective experience or focus, and time do not always allow them to attain immediacy, continuity, and complete objectivity (Bremmer and Nijs, 2020; Zheng, 2022). Artificial intelligence vocal classes convert speech signals processing capabilities and machine learning algorithms to multidimensional vocal performance analysis and provide learners with immediate feedback, specific feedback, and data-driven feedback in real time (Tajik, 2025; Liu and Guo, 2025; Wang and Fitri bin Haris, 2025). An example is real-time pitch trajectory display which enables learners to discover intonation deviations visually between the produced intonation and target melody line, and formant analysis which gives analytical data on the quality of vocal resonance (Takahashi et al., 2005).

According to the evidence available, such accurate feedback would greatly enhance efficiency of learning skills. Acknowledging that the research by Wang (2025) is an experimental design, the vocal learners with an AI feedback system showed a 40% greater rate of change to the accuracy of the pitches than the traditional control group. Nevertheless, current studies are mainly technical implementation studies and short-term behavioral studies, but little attention is given to the multidimensional nature of learning outcomes, especially very little attention is given to the long-term psychological impacts of learning outcomes. More importantly, the mediating mechanisms by which accurate feedback can regulate the learning outcomes in terms of shaping the psychological process of the learners have not been explored adequately. In itself, technology is not directly related to the generation of learning outcomes as it affects the cognitive, emotive, and metacognitive processes of learners (Drigas et al., 2023; Acosta-Gonzaga and Ramirez-Arellano, 2021). Thus, studying the psychological processes of how AI-generated accurate feedback affects the learning outcomes is essential toward the promotion of research in the field.

### The relationship between precise feedback, perceived competence, and learning outcomes in AI vocal courses

Perceived competence is one of the main ideas of the self-determination theory that is defined as a subjective assessment of the personal competence by a person in a certain field (Harter, 1982; Eccles and Wigfield, 2023; Wentzel et al., 2021). Perceived competence also appears in the form of subjective assessments of mastering the technical aspects of skills conversed during vocal training, including pitch-modal regulation, choral tone, and breath control. As per the cognitive evaluation theory, intrinsic motivation and behavioral performance depend on the environmental factors that contribute to the satisfaction of the ability to meet the need to perform competently in people (Fang et al., 2022; Zhang et al., 2024). The quality of the feedback offered by AI vocal courses could be one of the components of the environment that defines the perceived ability to master skills.

To begin with, the immediacy of the specific feedback reduces the length of the attempt-feedback loop and thus increases the speed of the positive reinforcement when learning a skill. In a situation whereby experiential learners give a voice exercise and subsequently get objective feedback on its efficiency, such feedback happens in time to enable them to develop the correct mental images of the skill within a very short period of time (Dunlosky et al., 2013). Second, evaluative ambiguity is minimized as the specificity and objectivity of the precise feedback makes the learners better understand the progress they are going through. In a conventional vocal training, the imprecise feedback by the teacher, e.g., tone is not rounded enough, when given to beginners tends to be confusion to them. A system based on AI, however, can give a quantitative and practical advice, e.g., raise the second formant by 200 Hz. The given specificity assists the learners to gain a clearer cognitive representation of their capabilities (López, 2025). Finally, the continuity of the accurate feedback makes it possible to view the visualization of micro-progress. This is done by performance data monitoring by AI systems over practice sessions which produce growth curves that expose otherwise invisible improvements. This techno-esthetic representation of advancement is a very vital channel toward the improvement of perceived mastery.

Extensive research evidence would be very helpful in the determination of the effect of perceived skill mastery on learning outcomes. Lehmann et al. (2007) discovered in the field of vocal music education that perceived skill command forecasts on continuity in practice, performance anxiety levels, and eventual success. The more perceived mastery of skill, the more high-level learners striving to meet the challenging goals and engage in deep thinking are more likely to become resilient to the challenge (Kucuksuleymanoglu, 2025). Therefore, AI-based accurate feedback might have an indirect positive impact on the learning results in the capacity to increase a perceived command of the skill, but this mediating role needs to be empirically substantiated.

### The relationship between precise feedback in AI vocal courses, self-efficacy, and learning outcomes

There is a fundamental concept of social cognitive theory, self-efficacy, which means that an individual is confident that he/she can effectively cope with particular actions (Bandura, 1997; Deng et al., 2025). Self-efficacy in vocal learning is observed through the confidence of the learners regarding their ability in mastering certain vocal tricks and playing the required repertoire. Bandura (1997) notes that there are four main sources of self-efficacy such as mastery experience, vicarious experience, verbal persuasion, and physiological and affective experiences. Accurate responses provided by AI vocal courses can have an impact on self-efficacy in a number of ways.

To start with, accurate feedback gives mastery experiences. By decomposing the compound dialogues into measurable and realizable sub-goals, the AI systems allow the learners to compound mastery experiences in a series of small-scale mastery successes—the strongest source of self-efficacy (Bandura, 1997). Second, the accuracy of the feedback makes the objectivity of precise feedback decrease evaluation-related anxiety. Traditional vocal teaching, on the one hand, is more likely to cause the social-evaluative anxiety of students due to teacher ratings, and this effect is especially likely to occur in the context of objective evaluations AI-based systems offer, thus providing a more secure psychological environment (Vistorte et al., 2024). Third, peer comparison data offered by some AI voice courses in anonymity give the vicarious experiences. Through seeing some positive progress in others with the same ability, the learners might also build confidence that they can also attain similar performance (Schunk and DiBenedetto, 2020).

Self-efficacy has been found to influence the results of learning to sing in the literature. Parncutt and G (2002) discovered that self-efficacy is a great predictor of quality of practice, success in competitions, and continuity in the practice among vocal students. Highly self-efficacy learners are more persistent in the face of difficult techniques and have a reduced degree of interference in performance situations of anxiety. It is worth noting that self-efficacy has not only behavioral but also cognitive outcomes such as goals, strategy, and the continued engagement in learning. Therefore, AI-based accurately provided feedback could indirectly promote learning goals by increasing self-efficacy; this mediating mechanism needs to be empirically proven.

### The mediating role of skill mastery in self-efficacy

Self-efficacy and mastering of skills are two very different constructs whereas they are theoretically related. Bandura (1997) argues that cognitive judgments about personal skills (conceptually related to skill mastery of perceived ability) are a very essential underpinning of self-efficacy judgments. In particular areas of learning, people initially create the impressions of their existing levels of skills (perceived skill mastery) and then assume that they are capable of overcoming related tasks in future (self-efficacy) (Schunk and DiBenedetto, 2020). When learning vocal music, it is necessary that the learner develops an objective assessment of his/her mastery of his/her skills including mastery of pitch, and the ability to support his/her breath. They subsequently use this evaluation of whether they are capable of learning a new song or study a new way of doing things. Such a psychological process, which involves going through the assessment of existing capability to a prediction of some success in the future, implies that what is perceived to be the mastery of skills can affect self-efficacy before it is well-developed.

Empirical evidence has been used to support this temporal relationship. In the field of medical education, Williams and Deci (1996) also noted that the perceived skill improvement was followed by the self-efficacy change and then the outcomes of learning. According to McCormick and McPherson (2003), in music education the perceived ability to master skills proved to be a significant predictor of self-efficacy, but in the new environment where AI is applied in learning vocabulary, it is not clear that perceived skill control and self-efficacy are a chaining mediating pathway. In particular, AI-assisted accurate feedback could initially stimulate the feeling of mastering the skills through objective and clear information about competencies of learners. This increased mastery can later lead to positive attributes of learners’ confidence in their future success in learning (self-efficacy) thus inevitably leading to better learning performance. Once confirmed, this chained mediation model would explain the overall psychological mechanism by which AI-driven accurate feedback can modify the learning outcomes: a multi-step process that will proceed to perception of an objective feedback and subsequently the ability itself (perceived skill mastery), the expectations of being successful in the future (self-efficacy), and subsequently the behavioral performance (learning outcomes).

This chained mediation model has a great theoretical interest in testing. Provided there is support, it would signify AI operated accurate feedback does not directly affect the learning outcomes but rather intervenes in a serial fashion, utilizing a cognitive appraisals and motivation anticipatory process. This observation would mean that the architecture of AI-driven vocal courses should not only make the feedback accurate but should also prioritize the optimization of the construction processes of the cognitive factors in learners by means of intentional feedback structure. In its application, it implies that AI systems must not just tell learners their present level of performance but also enable them to have positive expectations of improvement in the future.

### The present study: aims and implications

Building on the theoretical and empirical foundations discussed above, the present study aims to construct and empirically test a chained mediation model that clarifies the psychological mechanism through which AI-driven precise feedback influences vocal learning outcomes. Specifically, it seeks to investigate whether perceived skill mastery and self-efficacy serve as sequential mediators in this relationship, thereby disentangling the “cognitive–motivational” pathway that may underpin effective technology-enhanced vocal instruction. By integrating the self-determination theory and social cognitive theory within the context of AI-facilitated arts education, the study aspires to advance the theoretical understanding of how external technological affordances are transformed into internal psychological resources that drive learning.

The implications of this research are 2-fold. Theoretically, it extends feedback theory and technology-enhanced learning frameworks into the domain of skill-based artistic disciplines, providing a nuanced perspective on the sequential interplay between competence perception and efficacy beliefs. Practically, the findings are expected to offer actionable insights for the design and optimization of AI vocal courses, emphasizing that effective feedback systems should not only deliver technical precision but also scaffold learners’ perceived mastery and foster their self-confidence. Ultimately, this study responds to the call for a deeper integration of intelligent technology and psychological nurturing, contributing to the digital transformation of vocal education and serving as a reference for AI applications in other performance-based fields.

### Theoretical integration underlying the hypothesized model

**Figure 1:**
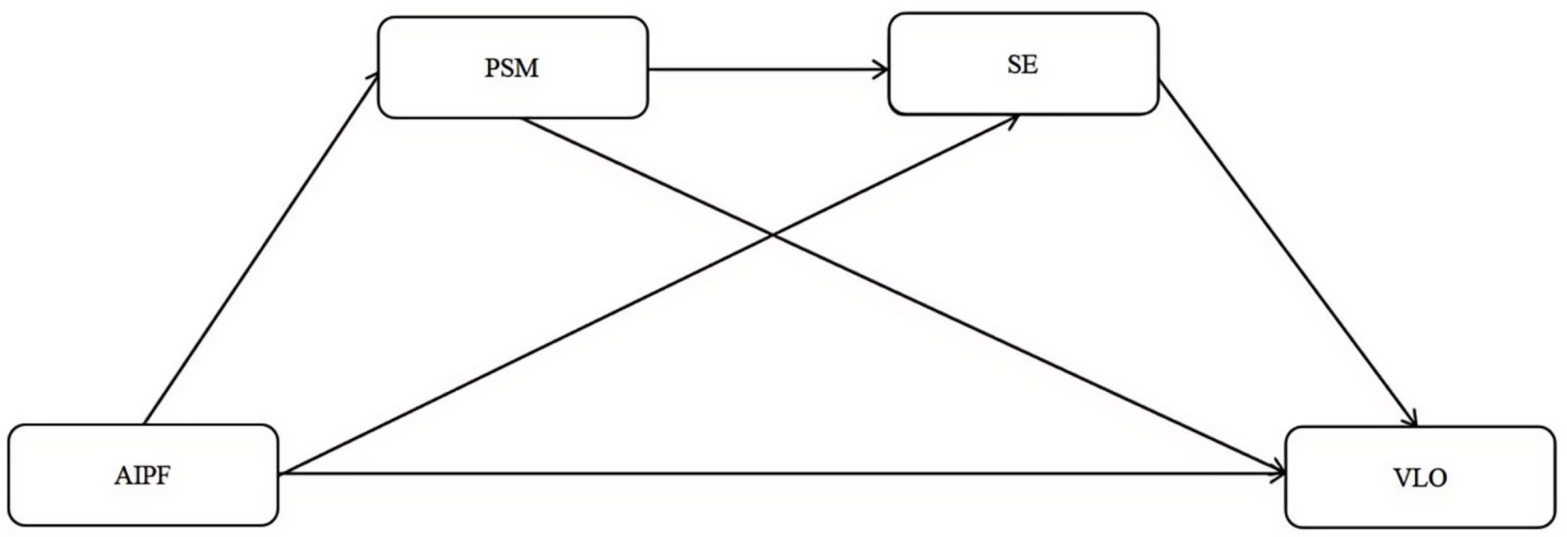
Schematic diagram of the research hypothesis model. AI-driven precise feedback (AIPF). Vocal learning outcomes (AIPF). Perceived skill mastery (AIPF). Self-efficacy (SE).

Before presenting the formal hypotheses, it is essential to integrate the theoretical perspectives that explicitly justify the relationships depicted in Figure 1. The proposed chain mediation model posits that AI-driven precise feedback influences vocal learning outcomes through a sequential cognitive–motivational process: perceived skill mastery and self-efficacy. This pathway is firmly grounded in two complementary frameworks—self-determination theory (SDT; Deci and Ryan, 2000) and social cognitive theory (Bandura, 1997).

First, the link between AI precise feedback and perceived skill mastery is rooted in cognitive evaluation theory, a sub-theory of SDT. According to this perspective, environmental events that satisfy individuals’ basic psychological need for competence enhance intrinsic motivation and perceived competence. AI feedback delivers objective, immediate, and highly specific performance data (e.g., pitch deviation values and formant characteristics), which enables learners to accurately monitor their ability levels and visibly track incremental improvements. This direct and unambiguous competence information acts as a powerful environmental affordance that bolsters perceived skill mastery (Fang et al., 2022; Zhang et al., 2024).

Second, the relationship between perceived skill mastery and self-efficacy is theoretically prescribed by Bandura’s (1997) assertion that self-efficacy judgments are fundamentally informed by cognitive appraisals of one’s own capabilities. In the context of vocal learning, when students perceive that they have mastered discrete technical skills (e.g., pitch accuracy and breath support), they are more likely to form positive expectations about their ability to succeed in future vocal tasks. This temporal and logical sequence—current ability perception preceding future efficacy expectation—has been empirically validated in educational settings (Williams and Deci, 1996; McCormick and McPherson, 2003). Thus, perceived skill mastery serves as a proximal cognitive precursor to the development of self-efficacy.

Third, the direct influence of AI feedback on self-efficacy can be explained through the primary sources of self-efficacy, particularly mastery experiences and physiological/affective states. By decomposing complex vocal techniques into achievable sub-goals and providing immediate success feedback, AI systems facilitate repeated mastery experiences, which are the most powerful source of self-efficacy. Simultaneously, the objectivity and non-evaluative nature of AI feedback reduce the social-evaluative anxiety commonly present in traditional vocal instruction, creating a psychologically safe environment that further strengthens efficacy beliefs (Vistorte et al., 2024; Schunk and DiBenedetto, 2020).

## Justifying the sequential order of mediators

We explicitly prioritize the “perceived skill mastery → self-efficacy” sequence over alternative models on both theoretical and logical grounds. Conceptually, the two constructs operate at distinct temporal loci: Perceived skill mastery is a retrospective and present-focused cognitive judgment of one’s current competence (“I have mastered this technique”), whereas self-efficacy is a prospective, future-oriented motivational belief about one’s capability to succeed in forthcoming tasks (“I will be able to perform this new piece”). Bandura’s (1997) social cognitive theory explicitly posits that self-efficacy beliefs are cognitively generated from the processing of efficacy-relevant information—most powerfully, from the cognitive interpretation of mastery experiences. That is, a learner must first perceive that they have succeeded (mastery) before this perception can be integrated into a generalized belief about future capability (efficacy). The alternative model—self-efficacy preceding perceived mastery—lacks this cognitive grounding, as it would require a forward-looking confidence belief to be formed prior to, or independent of, any cognitive assessment of actual competence. This is inconsistent with the fundamental tenet of self-efficacy theory, which holds that efficacy beliefs are not free-floating dispositions but rather cognitive products arising from the interpretation of performance information. Logically, in the context of an 8-week skill-acquisition intervention, the temporal flow is inherently structured: AI delivers real-time, granular performance data (e.g., “your pitch deviation is 12 cents”); the learner first processes this external feedback to form a cognitive appraisal of their current skill standing (perceived mastery); this solidified sense of “I can do this now” subsequently provides the inferential basis for the motivational belief “I can do this in the future” (self-efficacy). This “cognitive appraisal → motivational inference” pathway aligns with the broader social cognitive principle that cognition precedes and informs motivation in self-regulatory processes (Bandura, 1997; Schunk and DiBenedetto, 2020). Empirically, this temporal precedence has been documented in longitudinal studies across educational domains: Williams and Deci (1996) demonstrated in medical education that changes in perceived competence preceded and predicted changes in self-efficacy; McCormick and McPherson (2003) confirmed the same directional pathway in music education, where perceived ability was a significant antecedent of self-efficacy. While we acknowledge that in highly experienced populations or over longer timeframes, reciprocal dynamics may emerge (Bandura, 1997), the structured, novice-level, short-term nature of our vocal training intervention strongly favors the unidirectional “competence-to-confidence” sequence. Testing the reverse sequence would therefore be theoretically incongruent with the cognitive-developmental stage of our participants, who were systematically building competence perceptions from scratch before forming robust efficacy beliefs.

Taken together, the full sequential mediation model encapsulates a coherent “cognitive–motivational” mechanism: Externally provided AI feedback first shapes a learner’s cognitive self-appraisal (perceived skill mastery); this enhanced perception of competence, in turn, elevates the motivational belief of self-efficacy; and finally, heightened self-efficacy drives deeper engagement, persistence, and ultimately superior vocal learning outcomes. This integrated theoretical rationale directly informs each hypothesis outlined in the next section.

Taken together, the full sequential mediation model encapsulates a coherent “cognitive–motivational” mechanism: Externally provided AI feedback first shapes a learner’s cognitive self-appraisal (perceived skill mastery); this enhanced perception of competence, in turn, elevates the motivational belief of self-efficacy; and finally, heightened self-efficacy drives deeper engagement, persistence, and ultimately, superior vocal learning outcomes. This integrated theoretical rationale directly informs each hypothesis outlined in the next section.

### Research hypotheses

Based on the aforementioned theoretical analysis and literature review, this study formulates the following hypotheses (Figure 1):

*H1*: Precise feedback from AI vocal courses positively predicts vocal learning outcomes.

*H2*: Perceived skill mastery mediates the relationship between AI-driven precise feedback and vocal learning outcomes. Specifically, AI-driven precise feedback indirectly enhances vocal learning outcomes by increasing perceived skill mastery.

*H3*: Self-efficacy mediates the relationship between AI-driven precise feedback and vocal learning outcomes. Specifically, AI-driven precise feedback indirectly enhances vocal learning outcomes by strengthening self-efficacy.

*H4*: Perceived skill mastery and self-efficacy exert a sequential (chain) mediating effect in the relationship between AI-driven precise feedback and vocal learning outcomes. Specifically, AI-driven precise feedback first enhances perceived skill mastery, which subsequently strengthens self-efficacy, ultimately leading to improved vocal learning outcomes.

## Research methodology

### Research participants

The pretest-posttest experimental design with the experimental and control groups was used in this study. The initial sample was a convenience sample of 120 undergraduate students majoring in the field of music education in one of the universities. The inclusion criteria included the following: (1) similar background in foundational vocal skills (i.e., having passed the basic course assessment in vocals offered by the major); (2) a history of no previous professional vocal training (i.e., less than 1 year of systematic vocal training prior to admission); and (3) voluntary involvement with a signed informed consent. Three of all participants on the group dropped out due to personal reasons during the study and left the final valid sample to be 114 participants (57 in the experimental group and 57 in the control group). The number of participants was 36 male participants (31.6) and 78 female participants (68.4) aged between 18 and 22 years (*M* = 20.13, SD = 1.28). None of the participants were found to have any hearing loss and organically disordered vocal organs, and none of them was found to have any upper respiratory infection symptoms in the course of the last 1 week before the experiment.

### Intervention procedure

The 8-week intervention was designed to ensure comparability between the two groups in terms of lesson time, training repertoire, and instructor qualifications, with the only systematic difference being the mode of feedback delivery. Both groups attended two 90-min training sessions per week (totaling 24 h of instruction over the 8-week period) and were taught by the same certified vocal instructor. The complete training plan is detailed below.

### Experimental group: AI-based precise feedback training

Participants in the experimental group received training augmented by an intelligent vocal training platform equipped with a real-time acoustic analysis system. During each session, students performed vocal exercises and repertoire pieces while the AI system simultaneously captured their vocal output via a 16-bit/48 kHz professional sound card. The system provided real-time visual feedback on 12 vocal parameters (e.g., pitch accuracy, rhythmic stability, vowel purity, and register transition comfort) displayed on individual monitors. A typical training session consisted of the following:

Warm-up and basic vocal exercises (20 min): Students performed standardized breathing exercises, lip trills, and five-tone scale patterns. The AI system visually displayed the pitch trajectory of each note, with color-coded indicators (green for accurate, yellow for slightly deviated, and red for significantly off-pitch), enabling immediate self-correction.

Technical skill development (40 min): Each week focused on specific vocal techniques. For instance, in Week 3 (focused on legato phrasing), participants sang ascending and descending five-note scales while the AI system continuously tracked and displayed the smoothness of their pitch transitions, quantified as a “legato score” (0–100). Participants received individualized suggestions, such as “Maintain consistent airflow through the phrase transition; current airflow variance = 23%.”

Repertoire application (25 min): Students applied newly acquired techniques to assigned repertoire pieces. The AI system provided section-by-section analysis of pitch accuracy and rhythmic alignment, allowing learners to identify and rehearse problematic passages precisely.

Feedback review and goal setting (5 min): At the end of each session, the system generated a personalized progress report summarizing key performance metrics and comparing them to previous sessions. Participants set specific improvement goals for the next session (e.g., “Reduce average pitch deviation in the upper register from 18 cents to 12 cents”).

The week-by-week technical focus is summarized below:

- Week 1 (Foundation and baseline): Introduction to the AI platform; posture and natural breathing; basic pitch matching exercises; recording of pretest repertoire.
- Week 2 (Pitch accuracy): Intervallic accuracy training (minor/major seconds, thirds); real-time pitch trajectory visualization; immediate self-correction exercises.
- Week 3 (Legato and phrasing): Smooth pitch transition training; legato scoring with airflow consistency feedback; five-note ascending/descending scale exercises.
- Week 4 (Rhythm and tempo): Rhythmic precision exercises with metronomic AI feedback; complex syncopated patterns; ensemble timing exercises.
- Week 5 (Resonance and timbre): Formant tuning; resonance placement exercises; vowel modification in different registers with spectral envelope feedback.
- Week 6 (Dynamic control): Messa di voce exercises (gradual crescendo and decrescendo); visual intensity contour mapping; subglottal pressure estimation.
- Week 7 (Register transition): Smoothing the passaggio; real-time register transition analysis; exercises bridging chest and head voice.
- Week 8 (Integration and posttest): Comprehensive review and consolidation; mock performance with full AI analysis; recording of posttest repertoire.

### Control group: traditional instruction

The control group followed an identical schedule and curriculum in terms of repertoire and technical topics, but instruction was delivered in a conventional teacher-led format. During the 40-min technical skill development phase, the instructor-provided verbal feedback based on personal judgment and experience (e.g., “Your tone needs more roundness on the higher notes” or “Try to connect these two phrases more smoothly”). Piano accompaniment was used to guide pitch and rhythm. Feedback was not immediate and objective in the AI sense—rather, it depended on the instructor’s ability to observe, diagnose, and articulate suggestions without the support of real-time quantitative data. The control group did not have access to visual progress trajectories, and practice goals were set qualitatively in discussion with the instructor.

### Intervention fidelity

To ensure the reliability and consistency of the intervention across the 8-week period, a rigorous fidelity protocol was implemented. A trained research assistant, blind to the study hypotheses, attended 20% of all sessions (6 out of 32 sessions, selected randomly) for both groups. Using a standardized checklist, the assistant coded: (a) for the experimental group, the operational status of the AI system, the presence of real-time visual feedback, and the absence of instructor-provided technical vocal advice; (b) for the control group, the delivery of instructor-led verbal feedback and the absence of any AI-based visual feedback. Fidelity was calculated as the percentage of checklist items fully adhered to per observed session. Overall fidelity was 97.5% (SD = 4.1%), confirming the high integrity of the intervention implementation. Any minor deviations (e.g., a 2-min software restart) were documented and determined not to affect the overall treatment integrity.

The institutional ethics review committee of the university (Approval No. FZU-PSY2025-0302) approved the study protocol. Standardized tests on vocal skills and psychological scale tests were performed on all participants pretest and posttest.

### Research tools

#### AI vocal feedback system accuracy evaluation tool

This study employed an intelligent evaluation system based on acoustic parameter analysis as the AI feedback platform. Utilizing a 16-bit/48 kHz professional sound card for audio signal acquisition, the system applied real-time fundamental frequency detection algorithms and formant analysis techniques to generate instantaneous feedback and visual representations of core vocal parameters, including pitch accuracy, interval stability, and vowel purity (Wilson et al., 2008).

To evaluate feedback precision, the research team examined the correlation between system outputs and independent assessments conducted by three senior vocal experts using the Vocal Parameters Expert Criterion Scale (VPECS). The results demonstrated high consistency between the AI system and expert assessments across key dimensions, including pitch accuracy (*r* = 0.932, *p* < 0.001), rhythmic stability (*r* = 0.887, *p* < 0.001), and resonance quality (*r* = 0.851, *p* < 0.001). Furthermore, test–retest reliability analyses (involving the analysis of the same singing sample at 24-h intervals) revealed intraclass correlation coefficients (ICCs) ranging from 0.897 to 0.955 across all parameter assessments, indicating excellent measurement stability.

#### Multidimensional vocal learning outcome assessment scale

Learning outcomes were comprehensively evaluated using a self-developed instrument, the Multidimensional Vocal Learning Outcome Assessment Scale (MVLOAS). This scale comprises four dimensions—skill performance, knowledge comprehension, transfer application, and emotional engagement—with a total of 20 items.

The Skill Performance dimension was assessed through behavioral observation. Two blinded vocal experts independently scored participants’ pretest and posttest recordings of standardized repertoire pieces using the Vocal Technique Assessment Criteria (VTAC; *α* = 0.915). The Knowledge Comprehension dimension (six items) employed scenario-based judgment questions to assess participants’ understanding of vocal principles. The Transfer Application dimension (five items) required participants to demonstrate acquired techniques in unfamiliar repertoire. The Emotional Engagement dimension (four items) was measured using a 7-point Likert scale to assess attentiveness and enjoyment during the learning process.

The internal consistency reliability of the total scale was satisfactory (Cronbach’s *α* = 0.903). Confirmatory factor analysis (CFA) indicated good model fit (*χ*^2^/df = 2.137, RMSEA = 0.063, CFI = 0.941, TLI = 0.932, and SRMR = 0.041). Standardized factor loadings ranged from 0.621 to 0.856, with an average variance extracted (AVE) of 0.514 and composite reliability (CR) of 0.889, indicating satisfactory construct and convergent validity (Kline, 2016).

#### Vocal skill mastery perception questionnaire

Adapted from Williams and Deci’s (1996) Perceived Competence Scale, the Vocal Skill Mastery Perception Questionnaire (VSMPQ) was developed for application in vocal learning contexts. The questionnaire comprises four subscales—pitch control, breath management, timbre shaping, and emotional expression—with a total of 12 items (e.g., “I can accurately control my pitch”). Responses were rated on a 7-point Likert scale (1 = strongly disagree and 7 = strongly agree).

In this study, the internal consistency reliability of the total scale was satisfactory (Cronbach’s *α* = 0.884), with Cronbach’s α coefficients for the four subscales of 0.832, 0.819, 0.801, and 0.794, respectively. Confirmatory factor analysis (CFA) supported a four-factor structure with good model fit (*χ*^2^/df = 2.413, RMSEA = 0.058, CFI = 0.951, TLI = 0.943, and SRMR = 0.046). Standardized factor loadings ranged from 0.603 to 0.842, with an average variance extracted (AVE) of 0.527 and composite reliability (CR) of 0.901, indicating adequate convergent validity. Inter-factor correlations (0.412–0.587) were lower than the square root of each construct’s AVE, indicating satisfactory discriminant validity (Guay, 2022; Madigan and Kim, 2021).

#### Vocal learning self-efficacy scale

The Vocal Learning Self-Efficacy Scale (VLSES) was developed in accordance with Bandura’s (2006) guidelines for the construction of self-efficacy scales. The scale comprises two dimensions: Task Efficacy (8 items; e.g., “I believe I can master difficult vocal techniques”) and Coping Efficacy (6 items; e.g., “Even when practice is frustrating, I believe I can learn to sing well”), for a total of 14 items. Responses were rated on a 7-point Likert scale (1 = not at all confident and 7 = completely confident).

The internal consistency reliability of the total scale was excellent (Cronbach’s *α* = 0.912), with Cronbach’s α coefficients of 0.876 and 0.861 for the task efficacy and coping efficacy subscales, respectively. Confirmatory factor analysis (CFA) supported the two-factor structure, with acceptable model fit (*χ*^2^/df = 2.786, RMSEA = 0.066, CFI = 0.937, TLI = 0.925, and SRMR = 0.052). Standardized factor loadings ranged from 0.611 to 0.833, indicating adequate item reliability. The average variance extracted (AVE) for the task efficacy and coping efficacy dimensions was 0.511 and 0.503, respectively, with composite reliability (CR) coefficients of 0.874 and 0.866, indicating satisfactory convergent validity. Criterion-related validity analysis revealed significant positive correlations between the VLSES and Schunk and DiBenedetto’s (2020) revised Music Self-Efficacy Scale (*r* = 0.735, *p* < 0.001), as well as with actual vocal performance scores (*r* = 0.482, *p* < 0.001), indicating strong criterion-related validity.

### Data analysis

Data analysis in this research was done using IBM SPSS Statistics 26.0. To first provide an equivalent baseline on demographic features, pretest skills levels and psychological variables between the experimental and control groups, independent-samples *t*-tests, and chi-squared tests were applied to compare the results of the two groups before the intervention. Second, pretest, posttest, and paired-samples *t*-tests were carried out to test the difference in the results of voice learning outcomes, perceived mastery, and self-efficacy between pre-pretest and posttest within each group to identify the direct outcome of the intervention.

Third, to investigate mediating variables of perceived skill mastery and self-efficacy, a sequential (chain) mediation analysis was performed with the help of the PROCESS macro (Model 6) that Hayes Had designed and prepared (2018). In this analysis, the level of pretest of the dependent and mediating variables was controlled. The AI-enhanced specific feedback (coded as 1 = experimental group and 0 = control group) was defined as the independent variable, posttest vocal learning outcomes were determined as a dependent one, and the perceived skill mastery and self-efficacy in a sequence became mediators. The importance of indirect effects was determined by use of bias corrected percentile bootstrap with 5,000 resamples. The statistically significant indirect effects were regarded when the confidence interval did not include a value of zero. Two-tailed tests with a significance level of α = .05 were used for all analyses. The mediation model and standardized path coefficients are reported. To ensure the robustness of the results, the key variables were tested for normality, and bootstrap procedures were used to estimate the standard errors of the parameters where necessary.

### Pretest and posttest procedures

To ensure standardized and comparable data collection, all participants completed identical pretest and posttest assessments following the same administration protocol. The pretest was conducted during the week immediately preceding the commencement of the 8-week intervention, and the posttest was administered during the week immediately following the conclusion of the intervention.

Each testing session lasted approximately 90 min and was conducted in a dedicated university music laboratory equipped with calibrated recording equipment and individual testing stations. The assessment comprised three components administered in a fixed order:

1) Behavioral Performance Assessment (Skill Performance Dimension of MVLOAS). Participants were individually recorded performing two standardized vocal repertoire pieces—one classical Italian art song (e.g., “Caro mio ben”) and one Chinese art song—selected to assess a range of technical skills. The accompaniment was provided by a professional pianist following the same tempo markings for all participants. High-fidelity audio recordings (48 kHz, 24-bit) were captured using a matched pair of condenser microphones positioned at a standardized distance of 1.2 meters from the performer. These recordings were subsequently evaluated by two blinded vocal experts using the Vocal Technique Assessment Criteria (VTAC; *α* = 0.915). The experts were unaware of both group assignment and the testing time point (pretest vs. posttest), and the order of recordings was randomized to prevent systematic bias. The scores from the two raters demonstrated high inter-rater reliability (pretest ICC = 0.891; posttest ICC = 0.904), and the average of the two ratings was used as the skill performance score.
2) Self-Report Questionnaire Battery. Following the behavioral recording, participants completed a battery of self-report measures in a quiet classroom setting. The battery included the following:

The Multidimensional Vocal Learning Outcome Assessment Scale (MVLOAS)—specifically the Knowledge Comprehension (6 scenario-based items), Transfer Application (5 items), and Emotional Engagement (4 items) subscales, each rated on a 7-point Likert scale. The Skill Performance subscale was assessed behaviorally as described above.

The Vocal Skill Mastery Perception Questionnaire (VSMPQ; 12 items), assessing perceived mastery across four domains: pitch control, breath management, timbre shaping, and emotional expression. Responses were provided on a 7-point Likert scale ranging from 1 (strongly disagree) to 7 (strongly agree).

The Vocal Learning Self-Efficacy Scale (VLSES; 14 items), comprising the Task Efficacy (8 items) and Coping Efficacy (6 items) subscales. Responses were provided on a 7-point Likert scale ranging from 1 (not at all confident) to 7 (completely confident).

The total time required for completing the self-report battery was approximately 30–35 min. Standardized written instructions were provided on the cover page of the questionnaire booklet, and a trained research assistant, blind to group assignment, was present to clarify any procedural questions. No communication between participants was permitted during the testing session.

3) Demographics and Vocal Background. During the pretest only, participants completed a brief demographic form collecting information on age, gender, and prior vocal training history. At the posttest, this section was replaced with a brief open-ended item on perceived changes in learning experience (not included in the present quantitative analyses).

## Results

### Common method bias test

To manage the common method bias that may emerge due to self-report measures, the procedural controls were employed in the study design stage. These were partial reverse-coded items, anonymity, and multi-time–point data collection. Data analysis was further provided with the use of statistical procedures to carry out further tests.

The items of all self-report scales (e.g., perceived skill mastery, self-efficacy, and vocal learning outcomes) were analyzed using Harman single-factor test, which is an unrotated exploratory factor analysis. The findings showed that there were four factors having eigenvalues higher than one. The first common term explained 38.42% of the total variance which was less than the 40% critical value meaning that there was no one factor that was explaining the largest proportion of the variation.

To further confirm these results, thereafter, a confirmatory factor analysis (CFA) was performed based on the unmeasured latent method factor approach. Incorporating the model of the baseline measurement, another latent variable as common method variance was added, which made all measurement indicators to be simultaneously loaded on the construct factors and the method factor. Model comparisons showed that, when the method factor is taken into account, the model fit (*χ*^2^/df = 1.89, CFI = 0.972, TLI = 0.930, RMSEA = 0.068, and SRMR = 0.037) was higher than the one that the baseline model obtained (*χ*^2^/df = 2.31, CFI = 0.941, TLI = 0.930, RMSEA = 0.049, and SRMR = 0.05), but the average standardized loading of the variables that were observed to the method factor was only 0.128 and all variations in the correlation coefficients between the key constructs were less than 0.05. These outcomes show that the common method variance is not too great and does not represent a significant danger to the validity of the findings of the study. Generally, the data contained in this study do not show severe common method bias.

### Homogeneity test

To guarantee that the baseline level of the research is comparable between the experimental group and the control group before the intervention, this research performed tests of homogeneity on demographic variables, as well as the pre-intervention scores of core psychological pretests and vocal skills. An independent-samples *t*-test showed no significant differences in the experimental group and the control group with respect to age, pretest vocal learning outcome scores, pretest perceived skill mastery scores, or pretest self-efficacy scores (all *p* > 0.05). The chi-squared tests did not show any significant gender differences in the distribution between the two groups (*p* > 0.05). Detailed results are given in Table 1.

**Table 1:** Homogeneity test results for the experimental and control groups prior to intervention.

Test item	Experimental group (*n* = 57)	Control group (*n* = 57)	Statistic (*t*/*χ*^2^)	*p*
Age (years)	20.158 ± 1.301	20.105 ± 1.262	*t* = 0.214	0.831
Gender (male/female)	18/39	18/39	*χ*^2^ = 0.000	1.000
Vocal learning outcomes (pretest mean)	3.631 ± 0.411	3.661 ± 0.395	*t* = −0.404	0.687
Perceived skill mastery (pretest mean)	3.824 ± 0.951	3.912 ± 0.883	*t* = −0.507	0.613
Self-efficacy (pretest mean)	4.026 ± 0.896	3.982 ± 0.921	*t* = 0.257	0.797

### Pretest–posttest difference analysis

To examine the impact of AI-driven precise feedback on core variables, this study conducted paired-samples *t*-tests within both the experimental and control groups before and after the intervention. The results are presented in Table 2.

**Table 2:** Pretest–posttest differences between the experimental and control groups (*N* = 114).

Group	Variable	Pretest (*M* ± SD)	Posttest (*M* ± SD)	*t*	*p*	Cohen’s *d*
Experimental group (*n* = 57)	Vocal learning outcomes	3.631 ± 0.411	4.271 ± 0.358	−12.375	^<^0.001	1.638
	Perceived skill mastery	3.824 ± 0.951	5.263 ± 0.872	−11.694	^<^0.001	1.548
	Self-efficacy	4.026 ± 0.896	5.418 ± 0.765	−12.058	^<^0.001	1.596
Control group (*n* = 57)	Vocal learning outcomes	3.661 ± 0.395	3.851 ± 0.361	−2.674	0.009	0.354
	Perceived skill mastery	3.912 ± 0.883	4.228 ± 0.901	−2.023	0.047	0.268
	Self-efficacy	3.982 ± 0.921	4.158 ± 0.874	−1.433	0.155	0.190

The analysis indicated that the experimental group achieved significantly higher posttest scores than pretest scores across all three variables—vocal learning outcomes, perceived skill mastery, and self-efficacy (*p* < 0.001, Cohen’s *d* > 1.30). Effect sizes exceeded 1.50 for all variables, indicating very large effects. Among these, vocal learning outcomes exhibited the largest effect size (*d* = 1.638), followed by self-efficacy (*d* = 1.596), while perceived skill mastery also demonstrated a substantial effect (*d* = 1.548).

In contrast, the control group showed significant pretest–posttest differences only in vocal learning outcomes (*p* = 0.009), with a small-to-medium effect size (*d* = 0.354). Although perceived skill mastery improved, the effect size was small (*p* = 0.047, *d* = 0.268). No significant pretest–posttest difference was found for self-efficacy (*p* = 0.155, *d* = 0.190). These findings suggest that while traditional instructional methods may moderately enhance students’ vocal performance and perceived skill mastery, they have limited impact on strengthening confidence in completing vocal learning tasks (self-efficacy).

### Correlation analysis

To examine the interrelations between the core variables, Pearson’s forms of correlation between the posttest results of vocal learning outcomes, perceived skill mastery, and self-efficacy between the experimental and control groups were done separately. They are represented in Table 3.

**Table 3:** Correlation analysis results among core variables.

Variable	1	2	3
Experimental group (*n* = 57)			
1. Vocal learning outcomes	1		
2. Perceived skill mastery	0.712^**^	1	
3. Self-efficacy	0.684^**^	0.768^**^	1
Control group (*n* = 57)			
1. Vocal learning outcomes	1		
2. Perceived skill mastery	0.523^**^	1	
3. Self-efficacy	0.485^**^	0.543^**^	1

All variables of core in the experimental group had positive and significant correlations. Both perceived skill mastery (*r* = 0.712, *p* < 0.01) were strongly correlated with vocal learning outcomes and self-efficacy (*r* = 0.684, *p* < 0.01). Notably, the perceived mastery of skills and self-efficacy were found to be strongly associated (*r* = 0.768, *p* < 0.01), which meets the initial statistical condition of testing sequential (chain) mediation model.

There were also significant positive correlations between the variables in the control group, but the correlation coefficients were lower than those in the experimental group. It is worth noting that the correlation between perceived skill mastery and self-efficacy was significant (r = .543, p < .01), although it was weaker than that observed in the experimental group. Moreover, the correlations among vocal learning outcomes, perceived skill mastery, and self-efficacy were also stronger in the experimental group than in the control group.

These results indicate that the AI-guided specific feedback intervention could have enabled the interrelationships between these variables to become stronger, which led to a more unified skill-cognition-motivation framework.

### Sequential (chain) mediation model testing

To examine the sequential (chain) mediating roles of perceived skill mastery and self-efficacy in the relationship between AI-based precise feedback and vocal learning outcomes, hierarchical regression analysis was conducted. The results are presented in Table 4.

First, AI-based precise feedback (group membership) significantly and positively predicted vocal learning outcomes (β = .783, *p* < .001). Second, group membership significantly and positively predicted perceived skill mastery (β = .729, *p* < .001). Third, with self-efficacy as the dependent variable, both group membership (β = .453, *p* < .05) and perceived skill mastery (β = .439, *p* < .001) significantly and positively predicted self-efficacy.

Finally, when group membership, perceived skill mastery, and self-efficacy were simultaneously entered into the regression equation predicting vocal learning outcomes, perceived skill mastery (β = .387, *p* < .001) and self-efficacy (β = .323, *p* < .01) remained significant predictors, whereas the direct effect of group membership was no longer significant (β = .251, *p* > .05).

These findings indicate that perceived skill mastery and self-efficacy fully mediated the relationship between AI-based precise feedback and vocal learning outcomes.

**Table 4:** Sequential mediation effects of perceived skill mastery and self-efficacy.

Dependent variable	Predictor	*R*	*R* ^2^	*F*	*β*	*t*
Posttest vocal learning outcomes	Group (1 = experimental, 0 = control)	0.394	0.155	15.423^***^	0.783	3.927^***^
Posttest perceived skill mastery	Group	0.367	0.135	13.055^***^	0.729	3.613^***^
Posttest self-efficacy	Group	0.564	0.318	19.337^***^	0.453	2.339^*^
	Perceived skill mastery				0.439	4.503^***^
Posttest vocal learning outcomes	Group	0.684	0.468	24.050^***^	0.251	1.414
	Perceived skill mastery				0.387	4.008^***^
	Self-efficacy				0.323	3.310^**^

### Sequential (chain) mediation analysis: bootstrap test

**Figure 2:**
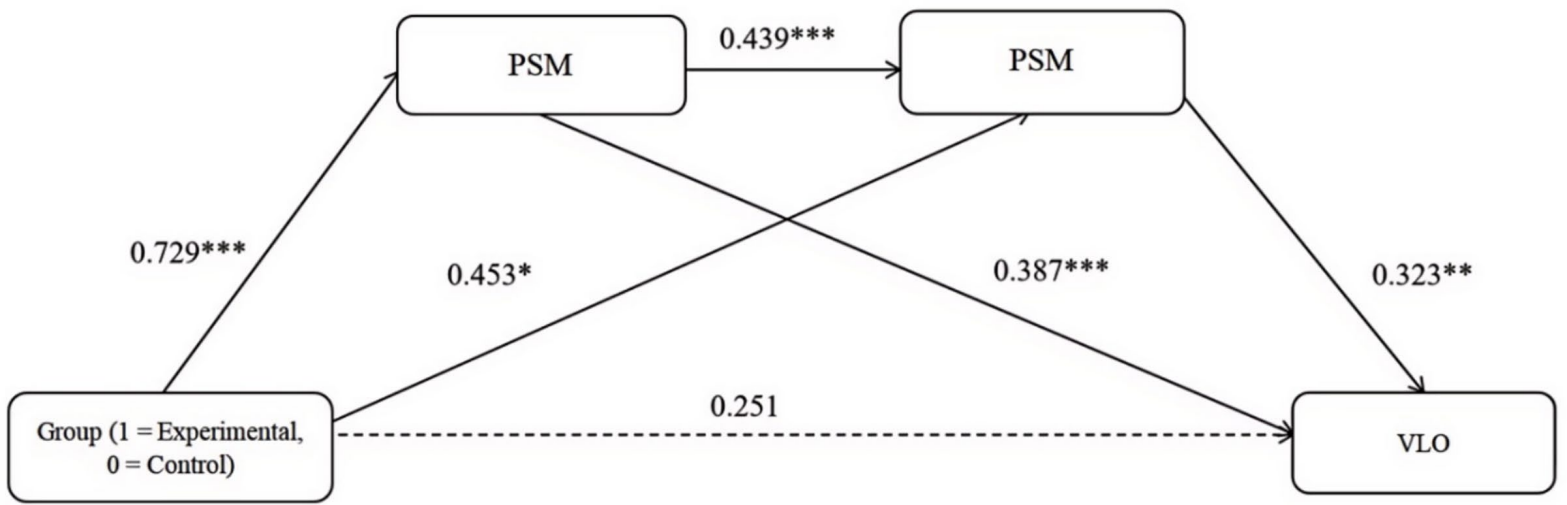
Validation model diagram.

Hierarchical regression analysis and bootstrap mediation analysis (PROCESS Model 6) were performed to investigate the sequential (chain) mediating roles of perceived skill mastery and self-efficacy among the connections between AI-driven precise feedback and vocal learning results. Table 4 and Figure 2 provide the results.

In the first place, predictive accuracy (vocal learning with subsequent use of AI-stimulated precise feedback and group membership) had significant and positive outcomes (783 = *p* < =human|). Second, group membership had a great influence and a positive significance in the perception of skill mastery (729 = −001). Third, group membership was reported to significantly predict self-efficacy in the instance that the latter was set as the dependent variable (0.453, *p* = 0.05). Self-efficacy was significantly and positively predicted by perceived skill mastery (= 0.439, *p* < 0.001).

Finally, in the event that group membership, perceived mastery of skills, and self-efficacy were all concurrently keyed into the regression model of predicting vocal learning outcomes, perceived skill mastery (= 0.387, *p* < 0.001) and self-efficacy (= 0.323, *p* < 0.01) were also a very important predictor, but the direct effect of group membership was non-significant (0251.332231, *p* > 0.05). What these results show is that the perceived skill mastery and self-efficacy mediated the effect between AI-precise feedback and vocal learning outcomes completely.

Bootstrap analysis with 5,000 resamples further confirmed the significance of the indirect effects. The total indirect effect was significant, whereas the direct effect was not, supporting a full sequential mediation model.

Table 5 presents the results of the bootstrap analysis testing the sequential (chain) mediating effects of perceived skill mastery and self-efficacy in the relationship between AI-driven precise feedback and vocal learning outcomes. The analysis indicated that the total effect of AI-driven precise feedback (group membership) on vocal learning outcomes was 0.777, with a 95% confidence interval that did not include zero, indicating a significant total effect. However, after incorporating the mediating variables, the 95% confidence interval for the direct effect included zero, suggesting that the direct effect was not statistically significant.

**Table 5:** Bootstrap test of the sequential mediation effects of perceived skill mastery and self-efficacy.

Path	Effect size	SE	95% CI lower	95% CI upper	Conclusion
Total effect	0.777	0.198	0.384	1.173	Established
Direct effect	0.249	0.176	−0.101	0.600	Not significant
Group → Perceived skill mastery → Vocal learning outcomes	0.280	0.119	0.065	0.524	Significant
Group → Self-Efficacy → Vocal Learning Outcomes	0.145	0.090	0.010	0.356	Significant
Group → Perceived Skill mastery → Self-efficacy → Vocal learning outcomes	0.103	0.076	0.016	0.310	Significant

Further analysis of the indirect effects revealed that all three mediation pathways were statistically significant. The indirect effect through perceived skill mastery (Group → Perceived Skill Mastery → Vocal Learning Outcomes) was 0.280. The indirect effect through self-efficacy (Group → Self-Efficacy → Vocal Learning Outcomes) was 0.145. Importantly, the sequential mediation pathway—Group → Perceived Skill Mastery → Self-Efficacy → Vocal Learning Outcomes—also demonstrated a significant indirect effect (effect = 0.103). In all cases, the 95% bootstrap confidence intervals did not include zero.

Collectively, these findings indicate that perceived skill mastery and self-efficacy fully and sequentially mediated the relationship between AI-driven precise feedback and vocal learning outcomes.

## Discussion

The design used in this research was pretest-posttest experimental design, alongside the experimental and control groups to statistically observe the role of AI-driven accurate feedback in vocal courses on learning the vocal learning outcomes, especially to examine the sequential (chain) mediating effect of perceived skill mastery and self-efficacy. The results were favorable to all hypothesized hypotheses.

First, the use of AI-based accurate feedback was helpful in improving vocal learning. The experimental participants showed a great superiority in the posttest performance in skill performance, knowledge comprehension, transfer application, and emotional engagement relative to the control group, which implies that AI-based feedback is an effective means to enhance multidimensional learning outcomes in vocal education.

Second, the mediation analysis showed that perceived skill mastery and self-efficacy were significant mediators in the association between AI-generated accurate feedback and the vocal learning outcome. Of most importance, the sequential mediating sequence of AI-based specific feedback/precise feedback to perceived mastery of skill-to-self-efficacy to vocal learning results showed a significant indirect but non-significant direct effect. This shows that the perceived skill mastery and self-efficacy singly compose a complete sequential medial process by which AI-based specific feedback affects the vocal learning outcome.

According to these findings, within this specific sample of novice undergraduate vocal learners and under the structured conditions of an 8-week AI-augmented course, AI-based vocal courses do not only have a positive impact on visible performance due to technological optimization but also allow the involved learner to progress to a higher level of learning by implicating sequential cognitive motivation mechanisms.

### The mechanism of AI vocal course precise feedback on learning outcomes

This research concludes that the facilitating effect of AI-motivated accurate feedback on the learning outcome of vocal is consistent with theoretical anticipations in terms of technology-enhanced learning. In terms of policy, the New Generation Artificial Intelligence Development Plan promoted by China specifically implies the promotion of the applications of AI in the whole process of teaching (Tu et al., 2019). The 14th Five-Year Plan of Digital Economic Development also notes that smart education is supposed to be focused on personalized development of talents (Shi, 2022). The example of AI vocal courses reflects this direction in the policies by incorporating AI-based specific feedback in the teaching formula. They reduce feedback delays and subjective bias of existing vocal teaching methods (e.g., fundamental frequency detection and formant tracking) by quantification of parameters such as pitch accuracy and rhythmic stability with real-time acoustic analysis algorithms (Lã and Fiuza, 2022; Zheng, 2022). An example of this is the use of Takahashi et al. (2005) trajectory visualization technology which offers the conceptual basis of the real-time pitch feedback in this study where learners would have been able to compare their performance with standardized values visually.

Second, immediate and specific feedback via AI generation produces the environment, which makes the visualization of the so-called micro-progress possible, thus, accelerating the process of learning a skill. Experimentally, Wang (2025) showed that AI feedback systems enhanced the accuracy of the vocal pitch improvements four times faster, which supports the significant improvement in the skills of the experimental group in the current study. Changes in this technological benefit lie in the reduction of the time of trial feedback that is in tandem with the principle of Dunlosky et al. (2013), which states that reinforcement is most effective when immediate. In addition, AI feedback can enhance objectivity, which decreases anxiety related to evaluation (Vistorte et al., 2024) because learners can concentrate more on the development of the necessary skills and not social comparison. This aligns with the findings of Acosta-Gonzaga and Ramirez-Arellano (2021) on the impact of emotional factors on the performance of learning. The AI-based specific feedback, therefore, directly increases the learning environment by its technological features: immediate, objective, and exhibited by the visualization, which, in its turn, indirectly improves the cognitive and affective interests of learners.

### Mediating role of perceived skill mastery

Perceived skill mastery was an important mediating variable in this study since it is a primary construct of the self-determination theory (Harter, 1982; Eccles and Wigfield, 2023). It has a strong mediating impact (= 0.387, *p* < 0.001) that AI-based precise feedback initially increases loyal voice competence of learners and thus leads to better learning results among learners. The result is justified by cognitive evaluation theory that argues that environmental feedback helps individuals to fulfill their desire to be competent, which in turn arouses intrinsic motivation (Fang et al., 2022; Zhang et al., 2024).

In vocal learning, AI systems encode the unclear vocal processes (e.g., rounder tone) as clear acoustic knowledge (e.g., increase the second formant by 200 Hz) and allow the learners to better perceive their progress and, in response, enhance their perceived skill mastery (López, 2025). In the current research, posttest perception of mastery of skill in the experimental group (M = 5.263) was significantly greater than perception of the control group (*M* = 4.228) and had a strong relationship with outcome of vocal learning (*r* = 0.712). These results are in line with the conclusion of Lehmann et al. (2007) that the perceived skill mastery is one of the major predictors of persistence and success in artistic learning.

Since it engages cognitive mechanism convergence, the continuity of AI-generated feedback visualizes the progress trajectories and makes it easier to attribute positive abilities in it. The accuracy of metacognitive monitoring is crucial to learning effectiveness according to the researchers (Dunlosky et al., 2013); AI feedback can improve the degree of performance evaluation accuracy in the self-assessment of learners because the technology offers objective performance data on these learners. This is in agreement with the Kucuksuleymanoglu (2025), achievement that the visualization of progress enhances resilience. In addition, the intermediary role of perceived skill mastery implies the transformation process between the external feedback and the internal cognition: Quantitative feedback offered by AI (e.g., pitch deviation values) is internalized as the confidence to the abilities (Williams and Deci, 1996) and eventually leads to adaptive learning behavior. In that way, the perceived skill mastery is not only a coincidence between the feedback provided by AI and the outcomes of vocal learning but also a key intermediary between the technological assistance offered outside and the psychological process occurring inside.

### The mediating role of self-efficacy

As a core construct in social cognitive theory (Bandura, 1997), self-efficacy exhibited a significant mediating effect in this study (*β* = 0.323, *p* < 0.01). This suggests that AI-driven precise feedback indirectly enhances learning outcomes by boosting learners’ confidence in their ability to perform future vocal tasks. This pathway validates Bandura’s (1997) theory of self-efficacy origins, wherein success/failure experiences, vicarious experiences, and emotional states collectively shape efficacy beliefs.

First, the AI system decomposes complex vocal skills into achievable sub-goals, allowing learners to accumulate “mastery experiences.” For example, the posttest self-efficacy scores in the experimental group (*M* = 5.418) were significantly higher than those in the control group (*M* = 4.158), with a large effect size (Cohen’s *d* = 1.596). This supports Deng et al.’s (2025) meta-analysis, which concluded that AI technology enhances learners’ success experiences through incremental progression design.

Second, the objectivity of AI feedback reduces evaluation anxiety, fostering a “psychologically safe” learning environment, as noted by Vistorte et al. (2024). This aligns with Schunk and DiBenedetto’s (2020) perspective that emotional regulation influences self-efficacy.

Notably, the mediating role of self-efficacy underscores the importance of motivational factors in technology-enhanced learning environments. Parncutt and G (2002) found that music learners with high self-efficacy were more likely to employ deep cognitive strategies and persist in challenging tasks. The experimental group’s superior performance in the transfer application dimension (e.g., singing unfamiliar pieces) exemplifies this mechanism. Furthermore, anonymized peer data provided by AI systems (e.g., progress curves of similarly skilled learners) may reinforce efficacy beliefs through “vicarious experience” (Schunk and DiBenedetto, 2020), aligning with Wentzel et al.’s (2021) meta-analysis, which found that peer comparison promotes academic achievement. Thus, the mediating pathway of self-efficacy suggests that AI-driven vocal courses function not only as skill-training tools but also as motivational systems. By enhancing learners’ “I can do it” beliefs, AI courses drive a shift in learning behavior from passive reception to active exploration.

### Chain mediation effect between skill mastery and self-efficacy

The main conclusion of the present research supports the sequential (chain) mediation effect among the mastering of a skill, self-efficacy and mastering a skills, self-efficacy, and finally mastering vocal learning outcomes. This chronological guide demonstrates the psychological cycle between the level of ability thought now to the anticipation of success in the future, which greatly amplifies the usage of technologies improved education in the era of the arts education.

Theoretically, this sequential mediation model confirms the position of Bandura (1997) in stating that self-efficacy judgments are founded on cognitive assessments of self-ability (i.e., skill mastery). In vocal training, students need to objectively evaluate their present competency (e.g., the competence of pitch control) to reasonably predict the future performance (e.g., learning new repertoire). This is quite consistent with the perceived ability efficacy performance chain signified by Williams and Deci (1996) as far as medical education is concerned.

The overall impact of the sequential mediation model (direct effects are not important) also shows that the impact of the AI-based feedback is essentially a process of gradual psychological construction. The AI technology makes performance data (e.g., pitch trajectory graphs) highly visible over time, to a learner, facilitating the process of forming correct perceptions of mastering a skill (Zhang et al., 2024). This is a cognitive base that is internalized to become certain that one can learn (Schunk and DiBenedetto, 2020). In the case of the example, the high correlation between self-efficacy and perceived skill mastery involved in this research confirms the conclusion made by McCormick and McPherson (2003) in music education that perceived ability is the predecessor of self-efficacy.

In practice, the sequential mediation model implies that AI-based vocal course development should consist of combining both the feedback about their current state (that is the ability to train) and the feedback about their potential improvement (the feature of the guidance or promise of improvement). As mentioned by Drigas et al. (2023), the future intelligent education must consider integrated interventions that cut across the domains of cognition-metacognition-affective, and this sequential model of mediation offers empirical evidence to this direction.

### Interpreting the magnitude of observed effect sizes

The standardized mean differences observed in this study (Cohen’s *d* = 1.638, 1.548, and 1.596 for learning outcomes, perceived mastery, and self-efficacy, respectively) are notably large, exceeding the typical range of *d* = 0.20–0.80 reported in meta-analyses of educational interventions (Kraft, 2020; Lortie-Forgues and Inglis, 2019). We address this directly to preempt concerns about robustness.

Why Such Strong Effects Emerged. Several design and contextual features likely contributed to these large effects. First, the intervention was unusually intensive: The experimental group received continuous, real-time AI feedback for 24 contact hours over 8 weeks. This dosage is substantially higher than many technology-enhanced learning studies where AI components supplement rather than replace core instruction. The immediacy and specificity inherent in the VocalCoach Pro 3.0 system—delivering millimeter-level feedback on pitch deviations, airflow variance, and formant structure within milliseconds—represents a fundamentally distinct pedagogical mechanism from delayed, qualitative human feedback. Second, the outcome measures were closely aligned with the intervention targets. The Multidimensional Vocal Learning Outcome Assessment Scale (MVLOAS) assessed precisely the vocal parameters that the AI system was designed to train, creating a tight conceptual coupling between intervention mechanism and measured outcome that can amplify standardized effect sizes. Third, the control condition—traditional, non-blind instructor-led instruction—provided a conservative but low-active baseline. The control group instructor, while experienced, could not replicate the immediacy, objectivity, or granularity of AI feedback, likely widening the contrast between conditions.

Potential Inflation Factors. We acknowledge factors that may have contributed to effect size inflation. Expectancy effects from unblinded participants in the experimental group—who were aware they were receiving a “technology-enhanced” intervention—may have inflated self-report measures (perceived mastery and self-efficacy). However, the behavioral skill performance dimension of the MVLOAS, rated by blinded vocal experts also yielded a very large effect (*d* = 1.638), suggesting that the observed effects are not solely artifacts of self-report bias. Common method variance, while not severe in our statistical tests (Harman’s single-factor = 38.42%), may still have modestly inflated correlations among the self-report mediators. Furthermore, the use of a convenience sample of motivated undergraduate music education majors—who had foundational vocal training and volunteered for the study—may have enhanced intervention responsiveness; these students were primed to extract maximal benefit from precise feedback.

Comparison with Similar AI-Based Intervention Studies. Although large, our effect sizes are not without precedent in the AI-assisted skill-acquisition literature. Wang (2025) reported a 40% greater rate of pitch accuracy improvement in an AI feedback group compared to traditional instruction—a relative gain that, when expressed as a standardized mean difference, corresponds to an effect size in the large-to-very-large range (*d* ≈ 1.0–1.4, depending on variance assumptions). Deng et al.’s (2025) meta-analysis of ChatGPT-enhanced learning found a pooled effect of *g* = 0.74 but noted that studies involving immediate, domain-specific, interactive feedback (as in our case) yielded effects substantially above the pooled estimate. In piano instruction, Wang (2025) observed large effects on self-efficacy (*d* ≈ 0.90) with a hybrid AI–human model less intensive than our fully AI-driven feedback system. Collectively, these comparisons suggest that our effect sizes, while at the upper bound, are plausible within the emerging “precision AI feedback” paradigm—a paradigm whose defining features (immediacy, specificity, continuity, and objectivity) depart meaningfully from the educational technologies typically represented in meta-analytic benchmarks. It is therefore appropriate to interpret our Cohen’s d values not as evidence of inflated results, but rather as indicators of a genuinely potent intervention when optimally implemented under well-controlled conditions.

We nevertheless recommend that future studies interpret these effect sizes with appropriate caution. Replication with larger, more heterogeneous samples, longer follow-up periods, and active comparison conditions (e.g., a sham AI condition with non-precise feedback) will be essential to determine whether the magnitude observed here is generalizable or specific to the current study’s unique combination of participants, context, and intervention intensity.

### Limitation and future research

Several limitations should be noted. First, the sample consisted of music education undergraduates from a single institution, which limits generalizability. The findings may not apply to learners of different ages, skill levels, or cultural backgrounds. Future studies should include more diverse samples to test the robustness of the model.

Second, the intervention lasted 8 weeks. While short-term effects were observed, it remains unclear whether these gains persist over time. Longitudinal designs with follow-up measurements are needed to examine the stability of perceived mastery, self-efficacy, and performance.

Third, some key variables were measured through self-report, which may introduce bias. Although performance was evaluated by experts, future research could combine behavioral indicators and objective measures to strengthen validity. In addition, different forms of AI feedback were not compared. Further work should explore how feedback design influences learning outcomes.

Finally, the proposed mediation pathway was not tested against alternative models, and causal direction cannot be firmly established. More rigorous designs, such as longitudinal or cross-lagged studies, would help clarify the relationships. Cultural factors may also play a role, and cross-cultural comparisons are needed.

## Theoretical and practical significance

The current research has strong theoretical implications and practical implication. On the one hand, the theoretically conceptualized study builds a chained mediation hypothesis, first, by applying the self-determination theory (Deci and Ryan, 2000) to the social cognitive theory (Bandura, 1997): AI feedback to sense of skill mastery to self-efficacy to learning effectiveness. This not only overcomes the rigidities of conventional technology-enhanced learning studies that typically put too much emphasis on behavioral evidence and expands our comprehension of the psychological processes that sit behind the learning of the artistic processes in AI contexts. Second, the study presents the notion of bringing specific feedback to the vocally educative sphere, pushing the limits of the feedback theory. Although in the past, AI feedback has been studied primarily in a cognitive style (e.g., Dunlosky et al., 2013), this study validates it to be as important in skill-based, emotionally intensive learning (e.g., vocal training) as it is in cognitive learning, a move that is supported by the idea by Parncutt and G (2002) that a specific emphasis on music learning activity must be established. In practice, this study is a contribution to the empirical basis of the construction and optimization of AI-based voice courses. First, it is stated that artificial intelligence systems should not be confined to the concept of technical precision but should be based on the principle of psychological empowerment. In particular, the feedback design ought to include progress visualization capabilities to increase the perceived control over a skill and self-efficacy at once (Wang, 2025). Second, to teachers, the results imply that the two methods can be used together to build blended strategies of teaching (i.e., emotional support of teachers and objective feedback of AI), which will be complementary (Bremmer and Nijs, 2020). The findings outlined by policymakers enable the introduction of the smart education policies of China and suggest to invest more into AI-driven arts education tools (Shi, 2022) and artificially intelligent teacher training to make educators prepare to become the objects in AI-enabled environments. Finally, the present research has wider implications to the digital transformation of education. The artificial intelligence (AI) in the precision feedback mechanisms can be extrapolated into other skill-based learning areas (e.g., sports and surgery), and the chained mediation model offers an overall scheme of self-discovery of the question of how technology can activate the potential in humans. According to the authors of the article, Drigas et al. (2023), the essence of the future school is a focus on achieving the synthesis of digital technology, metacognition, and emotional intelligence. This is the very integrative direction that all the practical importance of this study is oriented. With the adoption of a balance between technological empowerment and psychological nurturing, AI-powered vocal courses have the potential to serve as similar examples of the promotion of personalized, deep learning.

## Conclusion

The empirical findings of this study indicate that AI-specific feedback would be a great way to improve the results of vocal learning by rolling out a complete sequential mediational process comprising of perceived skill control and self-efficacy. Combining both the self-determination theory and social cognitive theory, the results indicate that AI feedback has an indirect effect on the performance through the initial buttressing of the current competence by the learners that leads to projected self-belief about achieving success in the future and eventually leads to effective learning. The findings contribute to the knowledge on the psychological processes of technology-enhanced learning. In practice, the design of AI vocal courses must shift the focus on technical accuracy to psychological empowerment, e.g., by embedding progress visualization in the course design, yet also developing blended models of instruction that involve the use of technology in teaching with human generated assistance. Although being inhibited by a rather mono-homogenous sample and a brief intervention period, this study will have a generalizable structure of AI-enhanced education and highlight that any successful technological empowerment should focus on mobilizing the inner psychological potential of learners to progress to personalized and profound learning.

## Data Availability

The raw data supporting the conclusions of this article will be made available by the authors, without undue reservation.
